# Regulated cell death and inflammasome activation in gut injury following traumatic surgery in vitro and in vivo: implication for postoperative death due to multiorgan dysfunction

**DOI:** 10.1038/s41420-023-01647-z

**Published:** 2023-11-07

**Authors:** Lingzhi Wu, Hailin Zhao, Mengxu Zhang, Qizhe Sun, Enqiang Chang, Xinyi Li, Wen Ouyang, Yuan Le, Daqing Ma

**Affiliations:** 1 Division of Anaesthetics, Pain Medicine and Intensive Care, Department of Surgery and Cancer, Faculty of Medicine, Imperial College London, Chelsea and Westminster Hospital, London, UK; 2 Department of Anesthesiology, Third Xiangya Hospital, Central South University, Changsha, 410013 Hunan PR China; 3 Hunan Province Key Laboratory of Brain Homeostasis, Third Xiangya Hospital, Central South University, Changsha, 410013 Hunan PR China; 4 Children’s Hospital, Zhejiang University School of Medicine, National Clinical Research Center for Child Health, Hangzhou, China

**Keywords:** Trauma, Translational research

## Abstract

Postoperative multi-organ dysfunction (MOD) is associated with significant mortality and morbidity. Necroptosis has been implicated in different types of solid organ injury; however, the mechanisms linking necroptosis to inflammation require further elucidation. The present study examines the involvement of necroptosis and NLR family pyrin domain containing 3 (NLRP3) inflammasome in small intestine injury following traumatic surgery. Kidney transplantation in rats and renal ischaemia-reperfusion (I/R) in mice were used as traumatic and laparotomic surgery models to study necroptosis and inflammasome activation in the small intestinal post-surgery; additional groups also received receptor-interacting protein kinase 1 (RIPK1) inhibitor necrostatin-1s (Nec-1s). To investigate whether necroptosis regulates inflammasome activity in vitro, necroptosis was induced in human colonic epithelial cancer cells (Caco-2) by a combination of tumour necrosis factor-alpha (TNFα), SMAC mimetic LCL-161 and pan-caspase inhibitor Q-VD-Oph (together, TLQ), and necroptosis was blocked by Nec-1s or mixed lineage kinase-domain like (MLKL) inhibitor necrosulfonamide (NSA). Renal transplantation and renal ischaemia-reperfusion (I/R) upregulated the expression of necroptosis mediators (RIPK1; RIPK3; phosphorylated-MLKL) and inflammasome components (P2X purinoceptor subfamily 7, P2X7R; NLRP3; caspase-1) in the small intestines at 24 h, and Nec-1s suppressed the expression of inflammasome components. TLQ treatment induced NLRP3 inflammasome, promoted cleavage of caspase-1 and interleukin-1 beta (IL-1β), and stimulated extracellular ATP release from Caco-2 cells, and MLKL inhibitor NSA prevented TLQ-induced inflammasome activity and ATP release from Caco-2 cells. Our work suggested that necroptosis and inflammasome interactively promote remote postoperative small intestinal injury, at least in part, through ATP purinergic signalling. Necroptosis-inflammasome axis may be considered as novel therapeutic target for tackling postoperative MOD in the critical care settings.

## Introduction

Postoperative death currently ranks as the 3^rd^ greatest contributor to death globally, with an estimated 4.2 million death occurring within 30 days after surgery each year [1]. Increasing clinical evidence suggested that surgery per se inflicted trauma and stress to the body to cause dysfunction of multiple organs (MOD) [2]. Postoperative MOD represents a formidable challenge to patient survival and quality of life, and greater effort is required to investigate underlying mechanisms and development strategies to prevent and treat MOD development following surgery to improve long-term outcome.

Notably, major noncardiac surgery is followed by dysfunction of multiple organs, encompassing the cardiovascular, pulmonary, gastrointestinal and renal systems, which is distant from the primary site of operation and is accompanied by systemic inflammation [3–6]. Hypoperfusion during/immediately after surgery is considered to be the initiating event in postoperative MOD, whereby multiple organs experience low flow perfusion to cause potential ischaemia and reperfusion injury in addition to traumatic cell death. Subsequently, death cells release proinflammatory cytokines and damage-associated molecular patterns (DAMPs) into the systemic circulation with potential dissemination to a distant site to cause remote organ injuries [7, 8]. Indeed, it has been well documented the onset of respiratory failure in acute kidney injury (AKI) patients, and the onset of renal dysfunction in lung injury patients [8]. The development of kidney, hepatic and cardiac injuries in Covid-19 pneumonia patients is another example of multi-organ cross-talk and highlights the difficulty in tackling MOD in the intensive care units [9]; post-surgical multiple organ injury may share such injurious crosstalk feature although the initiator (surgery vs virus) is totally different.

Necroptosis was reported as a key cell death mechanism underlying ischaemia-reperfusion organ injury [10, 11] and chronic inflammatory disease [12–14]. Our group previously demonstrated that necroptosis also plays a central role in remote lung injury that developed secondary to renal transplantation [15, 16]. The fact that inhibiting necroptosis prevents sterile (non-pathogen induced) inflammation suggests necroptosis as a form of “inflammatory” cell death, and highlights the therapeutic potential of targeting necroptosis in the prevention and treatment of multiple organ dysfunction and inflammation [17–19]. Necroptosis machinery was shown to lead to NLR family pyrin domain containing 3 (NLRP3) inflammasome activation within macrophages/bone marrow-derived monocytes in the context of autoimmune diseases (e.g., rheumatoid arthritis, dermatitis) [14, 20].

The involvement of necroptosis and inflammasome has been largely neglected in surgical setting. We consider the small intestine to be an important contributor to postoperative MOD, as the injured intestines may serve as a robust source of immunogenic mediators to precipitate and aggravate postoperative MOD [21]. In the present study, we aim to investigate the development of necroptosis and associated inflammasome pathway in the small intestine following a major surgery, and to further explore how necroptosis modulates inflammasome activity to contribute to MOD in in vitro and in vivo rats and mice models.

## Results

### Development of small intestine injury following ischaemic kidney transplantation

**Fig. 1:**
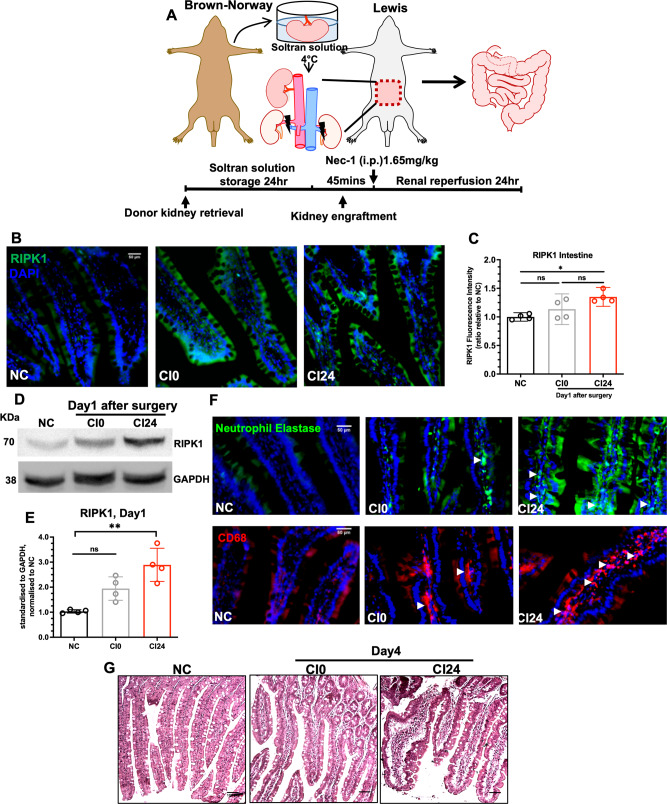
Ischaemic kidney transplantation is associated with remote small intestinal injury. **A** Illustration of ischaemic kidney allograft transplantation (CI24) workflow. **B** Immunofluorescence staining of RIPK1 in small intestine tissues from NC, CI0 and CI24 groups (magnification x20, scale bar 50 µm) at Day1. **C** Fluorescence intensity analysis of RIPK1 in small intestine (NC: 0.994 ± 0.047; CI0: 1.135 ± 0.168; CI24: 1.350 ± 0.104). **D** Western blot and (**E**) band intensity analysis of RIPK1 of small intestine samples (NC: 1.033 ± 0.067; CI0: 1.944 ± 0.469; CI24: 2.892 ± 0.661). **F** Immunofluorescence staining of neutrophil elastase and CD68 to label neutrophils and macrophages (arrowheads), respectively, in small intestine at Day1 (magnification x20, scale bar 50 µm). **G** Haemoxylin and eosin staining of small intestine tissues from NC, CI0, and CI24 groups at Day 4 (magnification x10, scale bar 100 µm). Data expressed as mean ± SD. NC vs. CI24: *P < 0.05, **P < 0.01; ns not significant; *n* = 4. Kruskal-Wallis test followed by Dunn’s post-hoc test.

On day1 after transplantation, immunofluorescence staining showed a significant increase in RIPK1 level in the small intestines in cold-ischaemia 24 h (CI24) cohorts (Fig.1B, C; *NC vs. CI24*, *P* = 0.024), but not in the cold-ischaemia 0 h group (live transplantation). Western blot corroborated a significant upregulation of the necroptosis mediator RIPK1 in the small intestines in the CI24 group (Fig. 1D, E; *NC vs. CI24*, *P* = 0.001; Supplementary File 1). Macrophage and neutrophil infiltrations into the small intestinal mucosa were also evident after transplantation (Fig.1F). Histology examination revealed minimal structural changes in the CI0 cohorts, whereas the CI24 Lewis recipients displayed villi blunting/deformation, mucosal oedema, and epithelial erosion/detachment (Fig. 1G). The collective findings suggest the development of remote injury and necroptosis in the small intestines following transplantation surgery, and upregulated expression of RIPK1 supports the use of RIPK1-selective inhibitor necrostatin-1s (Nec-1s) to block necroptosis and related pathways in the small intestines.

### Ischaemic kidney transplantation enhanced inflammasome activity in small intestine

**Fig. 2:**
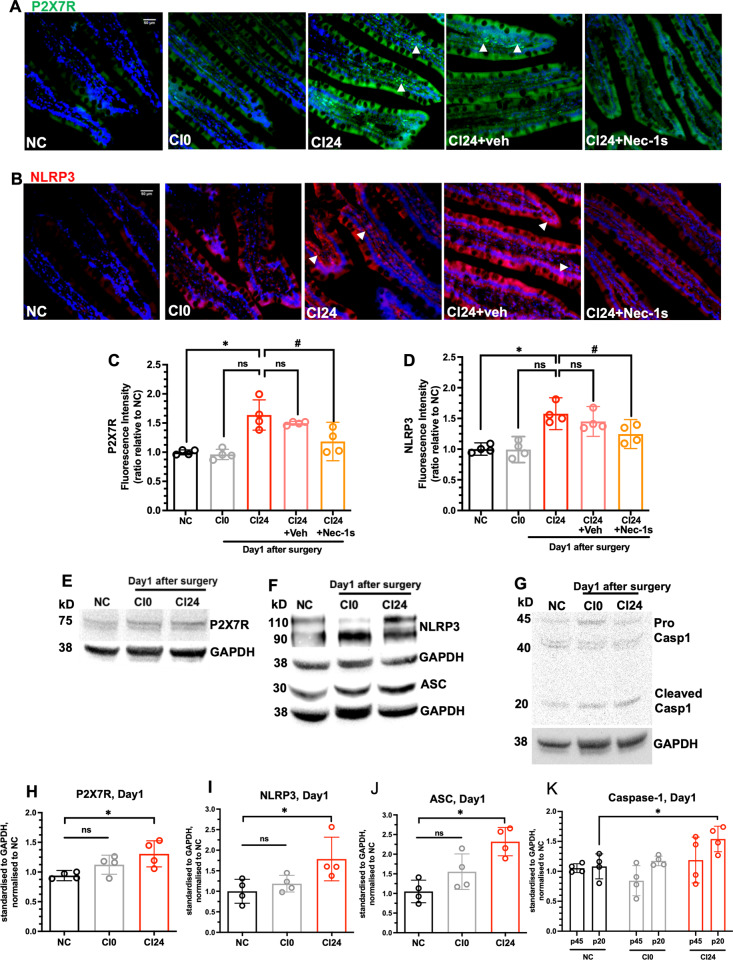
Ischaemic kidney transplantation leads to inflammasome activation in small intestine. **A** Immunofluorescence staining of P2X7R, and (**B**) NLRP3 from NC, CI0, CI24, CI24+veh and CI24+Nec-1s at Day1 (magnification x20, scale bar 50 µm); arrowheads indicate P2X7R or NLRP3 immuno-reactive cells. **C** Fluorescence intensity analysis of P2X7R (NC: 0.998 ± 0.042; CI0: 0.962 ± 0.089; CI24: 1.638 ± 0.260; CI24 + veh: 1.497 ± 0.030; CI24+Nec-1s: 1.183 ± 0.207). **D** Fluorescence intensity analysis of NLRP3 (NC: 1.002 ± 0.063; CI0: 0.993 ± 0.133; CI24: 1.577 ± 0.163; CI24 + veh: 1.451 ± 0.153; CI24 + Nec-1s: 1.246 ± 0.148). **E** Representative western blot of P2X7R, (**F**) NLRP3, ASC, (**G**) p45 and p20 cleaved caspase-1. **H** Band intensity analysis of P2X7R (NC: 0.941 ± 0.054; CI0: 1.124 ± 0.161; CI24: 1.307 ± 0.220). **I** Band intensity analysis of NLRP3 (NC: 1.000 ± 0.291; CI0: 1.186 ± 0.203; CI24: 1.786 ± 0.530). **J** Band intensity analysis of ASC (NC: 1.051 ± 0.288; CI0: 1.554 ± 0.453; CI24: 2.319 ± 0.360). **K** Band intensity analysis of p45 procaspase-1 (NC: 1.054 ± 0.074; CI0: 0.843 ± 0.252; CI24: 1.188 ± 0.383) and p20 cleaved caspase-1 (NC: 1.083 ± 0.208; CI0: 1.174 ± 0.080; CI24: 1.541 ± 0.213). Data expressed as mean ± SD. *NC vs. CI24: *P* < 0.05; CI24 vs. CI24+Nec-1s: ^#^*P* < 0.05; ns not significant; *n* = 4. Kruskal-Wallis test followed by Dunn’s post-hoc test.

The inflammasome is comprised of NLR family pyrin domain containing (NLRP3), apoptosis-associated speck-like protein containing a CARD (ASC) and pro-caspase 1, and upon recruitment to inflammasome pro-caspase1 is cleaved into the active form to enable downstream processing of IL-1β and IL-18 [22]. The canonical inflammasome activation pathway begins with ATP signalling through the purinergic receptor P2X7R [22, 23]. In CI24 cohorts, delayed kidney transplantation significantly increased the fluorescence intensities of P2X7R (Fig. 2A, arrowheads, and 2 C; *NC vs. CI24*, *P* = 0.018) and NLRP3 (Fig. 2B, arrowheads, and 2D; *NC* vs*. CI24*, *P* = 0.043) in the small intestines compared to NC, but not in the live transplantation CI0 group. Nec-1s treatment attenuated P2X7R immuno-reactive (Fig. 2A, C; *CI24 vs. CI24+Nec-1s*, *P* = 0.032) and NLRP3 immuno-reactive signals in the small intestines (Fig. 2B, D; *CI24* vs*. CI24+Nec-1s*, *P* = 0.032) from CI24 challenge. Western blot also showed significant upregulations of P2X7R (Fig. 2E, H; *NC* vs*. CI24*, *P* = 0.042; Supplementary File 2), NLRP3 (Fig. 2F, I; *NC* vs*. CI24*, *P* = 0.032; Supplementary File 3), ASC (Fig. 2F, J; *NC* vs*. CI24*, *P* = 0.013; Supplementary File 4) and cleaved caspase-1 p20 (Fig. 2G, K; *NC* vs*. CI24*, *P* = 0.044; Supplementary File 5) in the CI24 group. Our data demonstrated the activation of necroptosis and inflammasome pathway in the small intestines, and suggests that inhibiting necroptosis suppressed intestinal inflammasome activity following delayed kidney transplantation.

### Renal Ischaemia-reperfusion leads to necroptosis and inflammasome activation in the small intestines

**Fig. 3:**
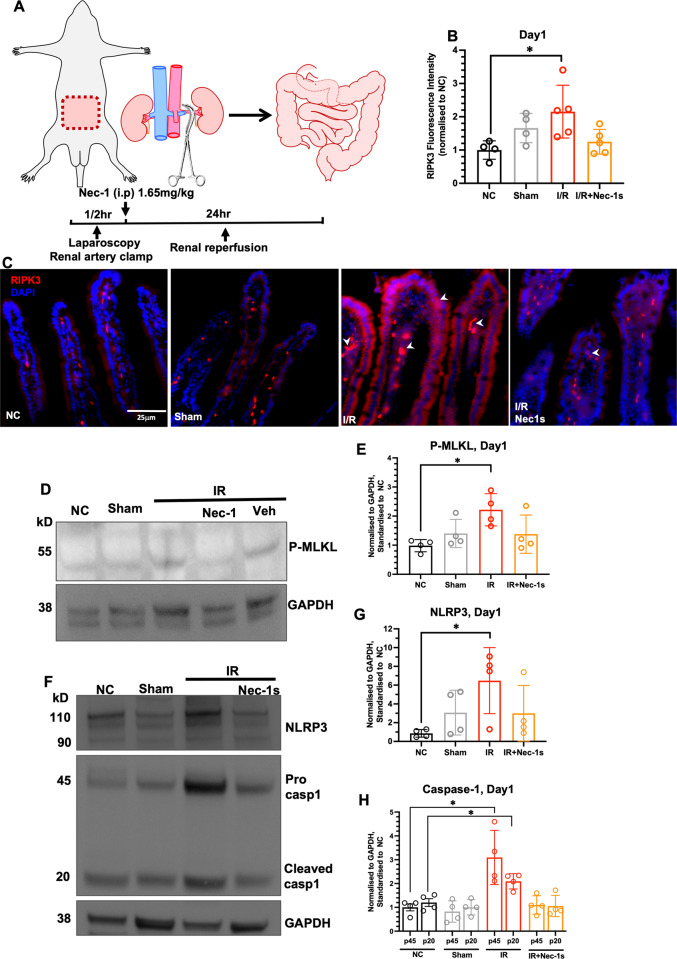
Renal ischaemia-reperfusion promotes necroptosis and inflammasome activity in small intestine. **A** Illustration of renal ischaemia reperfusion (I/R). **B** Fluorescence intensity analysis of RIPK3 in small intestines (NC: 1.000 ± 0.278; Sham: 1.661 ± 0.442; I/R: 2.153 ± 0.793; I/R+Nec-1s: 1.251 ± 0.372). **C** Immunofluorescence staining of RIPK3 of small intestine tissues, arrowheads indicate RIPK3-immunoreactive cells within villi (x40 magnification, scale bar 25 µm). **D** Representative western blot and (**E**) band intensity analysis of phosphorylated-MLKL on small intestines at Day1 (NC: 0.983 ± 0.210; Sham: 1.403 ± 0.484; I/R: 2.217 ± 0.554; I/R+Nec1s: 1.380 ± 0.658). **F** Representative western blot of NRLP3 (0.859 ± 0.406; Sham: 3.069 ± 2.384; I/R: 6.480 ± 3.520; I/R+Nec-1s: 2.999 ± 2.968), procaspase-1 (NC: 1.000 ± 0.291; Sham: 0.825 ± 0.450; I/R: 3.096 ± 0.568; I/R+Nec-1s: 1.101 ± 0.387) and cleaved caspase-1 (NC: 1.028 ± 0.153; Sham: 1.001 ± 0.323; I/R: 2.144 ± 0.232; I/R+Nec-1s: 1.057 ± 0.448) of small intestine tissues at Day1. **G** Band intensity analysis of NLRP3. **H** Band intensity analysis of procaspase-1 and cleaved caspase-1. All data expressed as mean ± SD. NC vs. I/R: *P < 0.05; *n* = 4–5. Kruskal-Wallis test followed by Dunn’s post-hoc test.

In a mice kidney ischemia-reperfusion model (Fig. 3A), the fluorescent intensity of necroptosis mediator RIPK3 was significantly increased in the I/R cohorts at day1 and was mostly localised to the epithelium and the underlying mucosae (arrowheads) when compared to that of naïve controls (NC) (Fig. 3B, C; *NC vs. I/R*, *P* = 0.011); Nec-1s treatment suppressed RIPK3 level to indicate prevention of necroptosis. The small intestine expression of phosphorylated MLKL (phos-MLKL) was also significantly increased in mice that underwent renal I/R but not sham surgery (Fig. 3D, E; *NC vs. I/R*, P = 0.045; Supplementary File 6), and Nec-1s treatment prevented CI24-induced MLKL phosphorylation. I/R also promoted inflammasome as evidenced by intestinal upregulations of NLRP3 (*NC vs. I/R*, P = 0.045), pro-caspase 1 (*NC vs. I/R*, *P* = 0.023; Supplementary File 7) and cleaved caspase-1 (*NC vs. I/R*, *P* = 0.046; Supplementary File 7), which were prevented in the presence of Nec-1s (Fig. 3F–H).

**Fig. 4:**
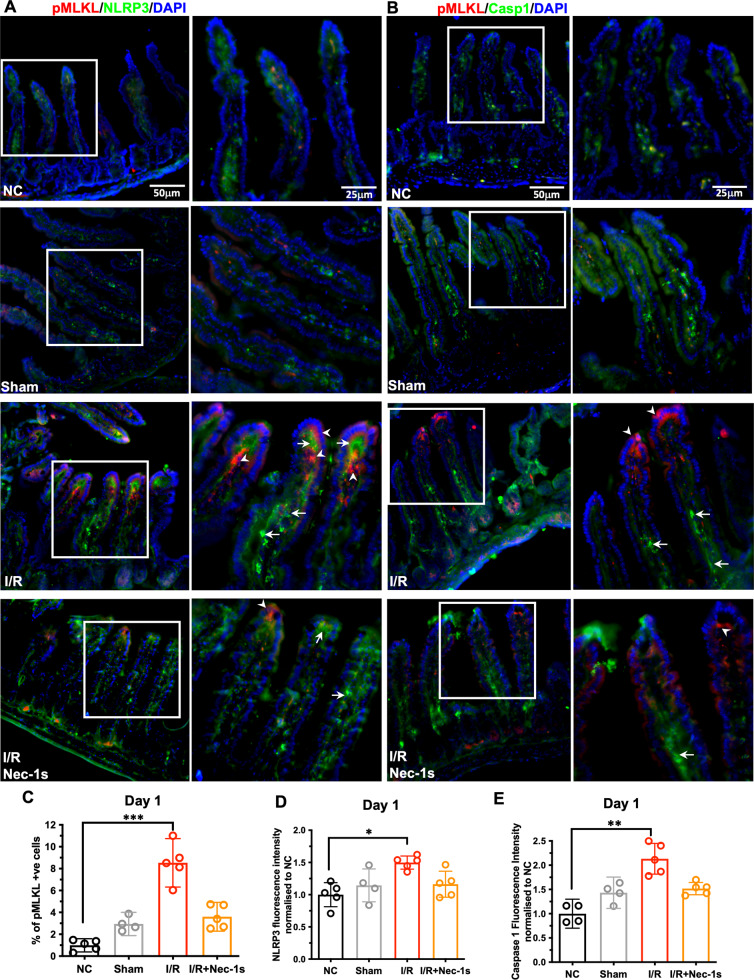
Visualisation of necroptosis and inflammasome activation in small intestine. **A** Double immunofluorescence staining of phosphorylated MLKL (red) and NLRP3 (green) or (**B**) caspase-1 (green) in NC, Sham, I/R and I/R+Nec-1s groups, boxed areas are enlarged to the right. Arrowheads indicate p-MLKL positive cells and arrows indicate NLRP3-immunoreactive cells (**A**) or caspase1-immunoreactive cells (**B**). Left panel, x20 magnification, scale bar 25 µm; right panel, x40 magnification, scale bar 50 µm. **C** Percentage of p-MLKL positive cells in small intestinal villi and mucosa (NC: 0.990 ± 0.490; Sham: 2.940 ± 0.667; I/R: 8.532 ± 1.783; I/R+Nec-1s: 3.600 ± 1.058). **D** Fluorescence intensity analysis of NLRP3 (1.000 ± 0.187; Sham: 1.144 ± 0.256; I/R: 1.498 ± 0.101; I/R+Nec-1s: 1.162 ± 0.202) and (**E**) caspase-1 (1.000 ± 0.189; Sham: 1.433 ± 0.203; I/R: 2.132 ± 0.317; I/R+Nec-1s: 1.517 ± 0.127) in small intestinal villi and mucosa. All data expressed as mean ± SD. NC vs. I/R: *P < 0.05, **P < 0.01, ***P < 0.001; *n* = 4. Kruskal-Wallis test followed by Dunn’s post-hoc test.

Small intestine samples were double-stained for phos-MLKL and NLRP3 or caspase-1 to localise necroptosis and inflammasome activity. Renal I/R significantly increased the percentage of phos-MLKL positive cells (Fig. 4A–C; *NC vs. I/R*, *P* < 0.001) that were concentrated at the intestinal villi tip (arrowheads), and to a lesser extent within the intestinal crypts. I/R also enhanced small intestinal NLRP3 (Fig. 4A, D; *NC vs. I/R*, *P* = 0.021) and caspase-1 (Fig. 4B, E; NC vs. I/R, *P* = 0.002) immunofluorescence intensities that were mostly concentrated in the underlying propia lamina. Nec-1s treatment ameliorated MLKL phosphorylation and attenuated I/R-induced NLRP3 and caspase-1 immunofluorescence intensities in the small intestines.

### Concurrent induction of necroptosis and inflammasome in small intestine epithelium-like cells

Previously reported necroptosis-inducing treatments included tumour necrosis factor-alpha (TNF-α) or lipopolysaccharides, along with cycloheximide (protein synthesis inhibitor), a SMAC (Second mitochondria-derived activator of caspase) mimetic (e.g. compound A) and/or a pan-caspase inhibitor (e.g. Z-VAD-FMK) [14, 20]. In the current study, we tested the necroptosis-inducing efficiency of a novel cocktail combination comprising TNF-α, the SMAC mimetic LCL-161 and the pan-caspase inhibitor Q-VD-Oph (abbreviated as TLQ). For comparison, another extra group of cells was treated with TNF-α and LCL-161 (abbreviated as TL) to activate both apoptosis and necroptosis.

**Fig. 5:**
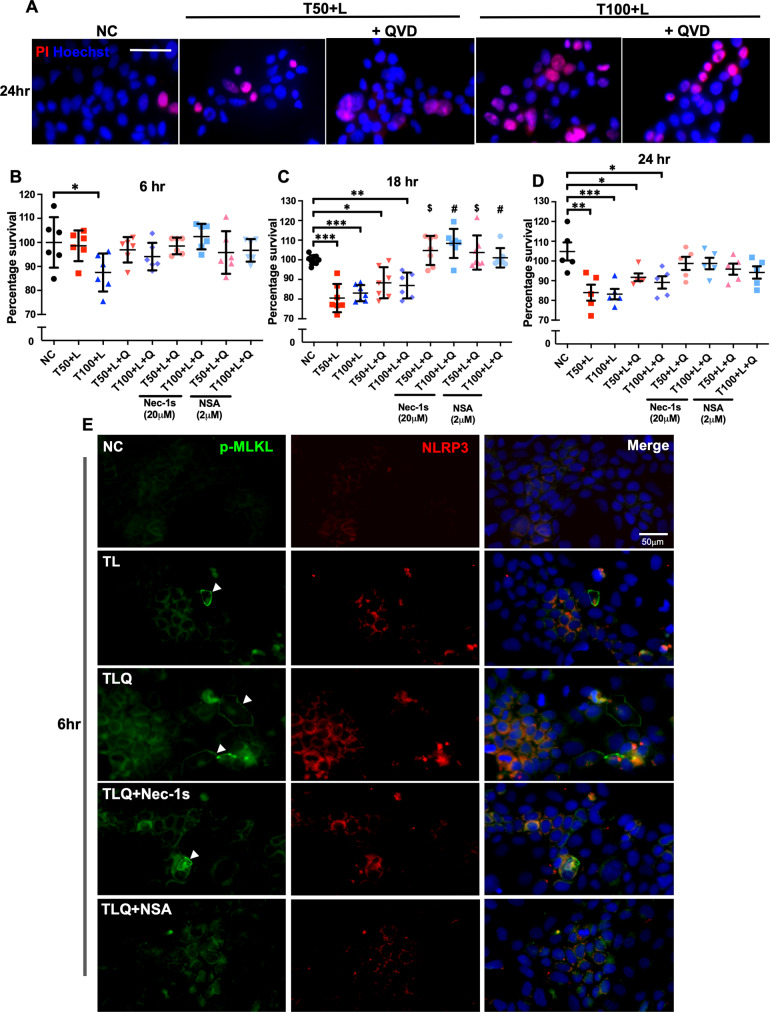
Induction of necroptosis in Caco-2 cells. **A** Propidium iodide and Hoechst staining of cells 24 h after indicated treatments. **B**–**D** CCK-8 assay on Caco-2 cell viability at different times after indicated treatments, expressed as % change from NC. Data expressed as mean ± SD. 6 h – NC: 1.000 ± 0.105; T50 + L: 0.986 ± 0.064; T100 + L: 0.875 ± 0.079; T50 + L + Q: 0.969 ± 0.053; T100 + L + Q: 0.941 ± 0.057; T50 + L + Q+Nec-1s: 0.985 ± 0.035; T100 + L + Q+Nec-1s: 1.024 ± 0.053; T50 + L + Q + NSA: 0.958 ± 0.089; T100 + L + Q + NSA: 0.967 ± 0.047. [*P < 0.05 vs. NC]; *n* = 6. 18 h – NC: 1.000 ± 0.023; T50 + L: 0.805 ± 0.072; T100 + L: 0.831 ± 0.041; T50 + L + Q: 0.883 ± 0.080; T100 + L + Q: 0.870 ± 0.066; T50 + L + Q+Nec-1s: 1.048 ± 0.074; T100 + L + Q+Nec-1s: 1.083 ± 0.074; T50 + L + Q + NSA: 1.037 ± 0.087; T100 + L + Q + NSA: 1.010 ± 0.050. [*P < 0.05, **P < 0.01 and ***P < 0.001 vs. NC; ^$^*P* < 0.05 vs. T50 + L + Q; ^#^*P* < 0.05 vs. T100 + L + Q]; *n* = 7–9. 24 h – NC: 1.048 ± 0.103; T50 + L: 0.839 ± 0.091; T100 + L: 0.832 ± 0.059; T50 + L + Q: 0.917 ± 0.044; T100 + L + Q: 0.981 ± 0.70; T50 + L + Q+Nec-1s: 0.988 ± 0.075; T100 + L + Q+Nec-1s: 0.987 ± 0.065; T50 + L + Q + NSA: 0.959 ± 0.063; T100 + L + Q + NSA: 0.942 ± 0.070. [*P < 0.05, **P < 0.01 and ***P < 0.001 vs. NC]. *n* = 5. One-way ANOVA followed by Tukey’s post-hoc test. **E** Representative immunofluorescence images of phos-MLKL (green) and NLRP3 (red) in Caco2 cells at 6 h. Arrowheads indicate phos-MLKL positive cells. Magnification x40, scale bar 50 µm.

Both TL and TLQ triggered Caco-2 cell death seen as increased propidium-positive staining (Fig. 5A) and reduced CCK8 cell viability (Fig. 5B–D), when TNF-α was given at 50 ng/mL (T50) or 100 ng/mL (T100) in the treatment cocktail. TL or TLQ-induced cell death was more prominent at 18 h and 24 h (Fig. 5D; NC vs. T100 + L + Q, *P* = 0.038; NC vs. T50 + L, *P* = 0.002; NC vs. T100 + L, *P* = 0.001), whereas at 6 h (Fig. 5B) only T100 + L significantly reduced cell survival (NC vs. T100 + L, *P* = 0.048). RIPK1-specific inhibitor Nec-1s (20 µM) or MLKL-specific inhibitor necrosulfonamide (NSA, 2 µM) improved cell viability in TLQ-treated groups, to support TLQ as a novel cocktail treatment that selectively induced necroptosis in Caco-2 cells. For all subsequent studies TNF-α was used at 100 ng/mL in the different treatment cocktails.

**Fig. 6:**
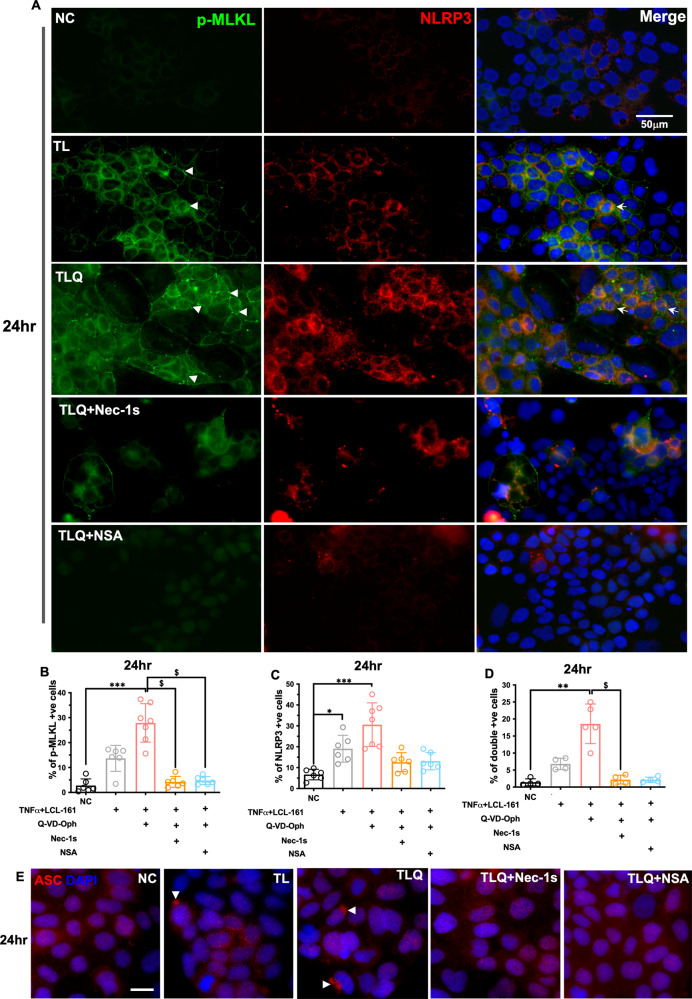
Necroptosis is associated with inflammasome activation in Caco-2 cells. **A** Representative photomicrographs of phos-MLKL (green) and NRLP3 (red) immunostaining in Caco-2 cells 24 h after indicated treatments. Arrowheads indicate pMLKL-positive cells (left panel), and arrows indicate pMLKL and NLRP3-double positive cells (right panel). Magnification x40, scale bar 50 µm. **B** Percentage of p-MLKL positive cells (NC: 2.738 ± 2.669; TL: 13.67 ± 5.210; TLQ: 27.89 ± 7.725; TLQ+Nec-1s: 4.213 ± 2.212; TLQ + NSA: 4.753 ± 1.631); *n* = 6–7. **C** Percentage of NLRP3 positive cells (NC; 6.680 ± 2.538; TL: 19.08 ± 6.450; TLQ: 30.57 ± 10.43; TLQ+Nec-1s: 12.62 ± 4.567; TLQ + NSA: 13.10 ± 4.143); *n* = 6–7. **D** Percentage of pMLKL and NLRP3 double positive cells (NC: 1.430 ± 1.014; TL: 6.825 ± 1.696; TLQ: 18.59 ± 5.820; TLQ+Nec-1s: 2.193 ± 1.293; TLQ + NSA: 2.143 ± 0.793); *n* = 4–5. **E** Immunofluorescence staining of ASC in Caco-2 cells at 24 h, arrowheads indicate speck-like ASC distribution in the cytoplasm. Magnification x63, scale bar 20 μm. All data expressed as mean ± SD. NC vs. TL: *P < 0.05; NC vs. TLQ: **P < 0.01, ***P < 0.001; TLQ vs. TLQ+Nec-1s: ^$^*P* < 0.05; TLQ vs. TLQ + NSA: ^$^*P* < 0.05. Kruskal-Wallis test followed by Dunn’s post-hoc test.

As early as at 6 h after treatments (Fig. 5E), moderate phosphorylation of MLKL was seen in TLQ-treated Caco-2 cells, where phos-MLKL immunoreactive signals localised to the plasma membrane (arrowheads), in line with previous studies reporting that MLKL translocates to the plasma membrane to cause perforation and cell death [24–26]. NLRP3 immunofluorescence was primarily seen in cytoplasm and the intensity was increased by TL or TLQ treatment at 6 h (Fig. 5E). At 24 h, MLKL phosphorylation (Fig. 6A, B; % of pMLKL+ve cells, NC vs. TLQ, *P* < 0.001) and NLRP3 immunofluorescence intensity (Fig. 6A, C; NC vs. TL, *P* = 0.032; NC vs. TLQ, *P* < 0.001) were significantly elevated in TLQ-treated cells. TLQ also significantly increased the number of phos-MLKL and NLRP3 double positive cells (Fig. 6D; NC vs. TLQ, *P* = 0.005). Phos-MLKL and NLRP3 co-localisation suggests co-activation of necroptosis and inflammasome within the same intestinal epithelial cell, whereas singly stained cells support that necroptosis and inflammasome are activated in different, neighbouring cells. Moreover, as inflammasome activation is characterised by the nuclear-to-cytoplasm translocation of ASC and formation of cytoplasmic ASC speck [27], we have shown that TLQ and to lesser extent TL, induced cytoplasmic ASC aggregation in Caco-2 cells compared to NC (Fig. 6E, arrowheads).

### Necroptosis inhibition suppressed inflammasome activity in mono- and co-cultures

**Fig. 7:**
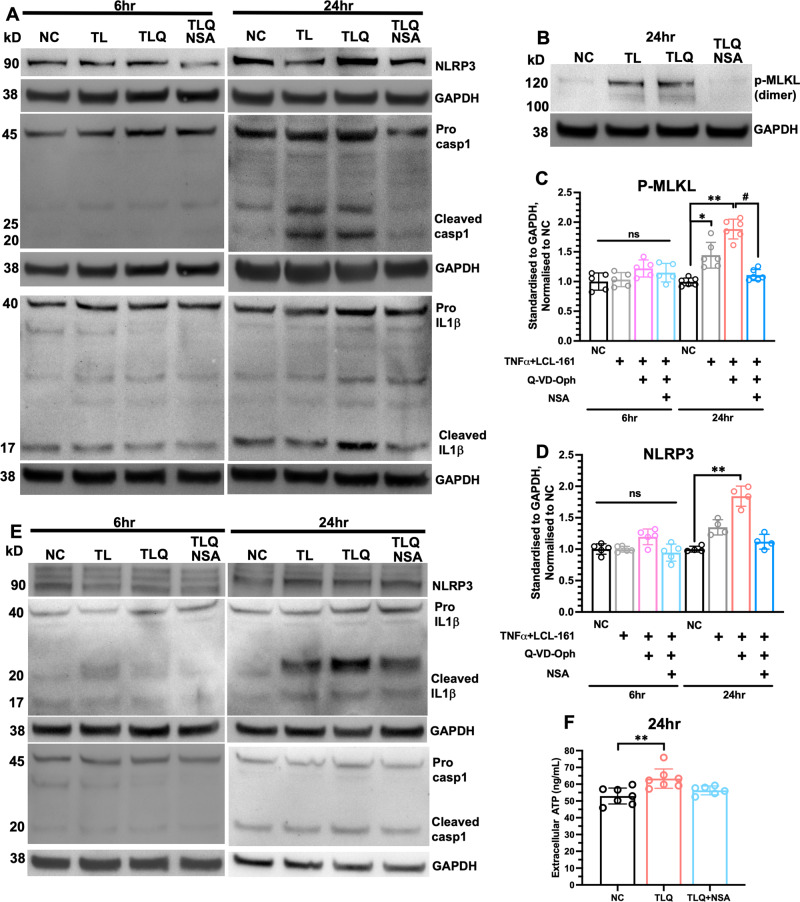
Inhibition of necroptosis prevents inflammasome activation in vitro. **A**, **B** Representative western blot of phos-MLKL, NLRP3, pro/cleaved caspase-1 and pro/cleaved IL-1β in Caco2 mono-culture 6 h and 24 h after indicated treatments. **C** Band intensity analysis of phos-MLKL in Caco-2 cells (NC: 0.995 ± 0.066; TL: 1.443 ± 0.217; TLQ: 1.883 ± 0.167; TLQ+Nec-1s: 1.109 ± 0.094); *n* = 6. **D** Band intensity analysis NLRP3 in Caco-2 cells (NC: 1.000 ± 0.046; TL: 1.348 ± 0.121; TLQ: 1.843 ± 0.161; TLQ+Nec-1s: 1.118 ± 0.118); *n* = 4. **E** Representative western blot of inflammasome components in Caco-2 plus U937 co-culture 6 h and 24 h after indicated treatments. **F** Extracellular ATP content from Caco-2 cells 24 h after indicated treatments. All data expressed as mean ± SD. NC vs. TL: *P < 0.05; NC vs. TLQ: **P < 0.01; TLQ vs. TLQ + NSA: ^#^*P* < 0.05; ns not significant. Kruskal-Wallis test followed by Dunn’s post-hoc test.

Immunofluorescence staining revealed that Nec-1s or NSA prevented TLQ-induced MLKL phosphorylation (Fig. 6A, B; TLQ vs. TLQ+Nec-1s, *P* = 0.012; TLQ vs. TLQ + NSA, *P* = 0.026), diminished NLRP3-positive (Fig. 6A, C; TLQ vs. TLQ+Nec-1s, *P* = 0.067; TLQ vs. TLQ + NSA, *P* = 0.081) and phos-MLKL/NLRP3 double-positive cells at 24 h (Fig. 6A, D; TLQ vs. TLQ+Nec-1s, *P* = 0.039; TLQ vs. TLQ + NSA, *P* = 0.082) and decreased ASC aggregation (Fig. 6E), to suggest that necroptosis signalling is upstream to inflammasome activation. Western blot data corroborated such findings in Caco-2 monoculture, whereby TLQ induced MLKL phosphorylation (NC vs. TLQ, *P* < 0.001) and inflammasome components at 24 h, including NLRP3 (NC vs. TLQ, *P* = 0.004), pro/cleaved caspase-1 and cleaved IL-1β (Fig. 7A–D; Supplementary Files 8, 9). Treatment with NSA prevented MLKL phosphorylation (TLQ vs. TLQ + NSA, *P* = 0.017), downregulated NRLP3 (TLQ vs. TLQ + NSA, *P* = 0.085), and prevented pro-caspase1 and pro-IL-1β cleavage (Fig. 7A; Supplementary File 8). TLQ combination also increased inflammasome activation in Caco2/U937 co-culture at 24 h and NSA inhibited cleavage of caspase-1 and IL-1β (Fig. 7E; Supplementary File 10). Given that ATP signalling through purinergic receptor P2X7R initiates the assembly and activation of NLRP3-inflammasome, we investigated whether necroptosis may regulate inflammasome through ATP/P2X7R axis. In Caco-2 cells, TLQ significantly increased the extracellular ATP content (Fig. 7F, NC vs. TLQ, *P* = 0.005) and NSA treatment decreased extracellular ATP to level comparable to that of NC. Collectively, our data suggest increased ATP release from necroptotic cells promoted inflammasome activity, and inhibiting MLKL-mediated necroptosis prevented ATP release and subsequent inflammasome activation.

## Discussion

**Fig. 8:**
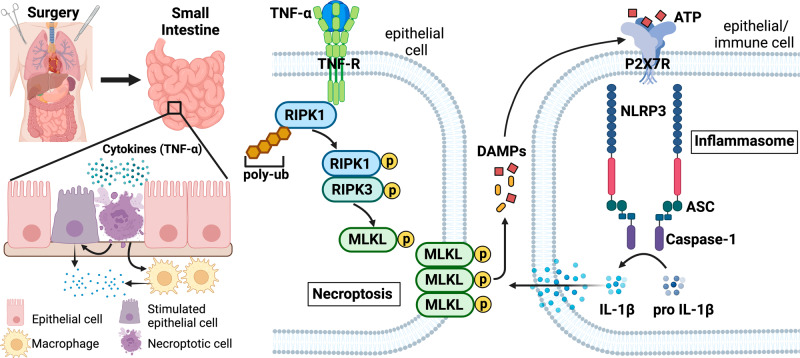
Putative mechanisms underlying postoperative small intestinal injury. Surgical trauma is associated with systemic increase in pro-inflammatory cytokines (TNF-α) to lead to necroptosis of small intestinal epithelial cells. Stimulation of TNF receptor and de-ubiquitination are required for the activation of RIPK1 and the subsequent phosphor-activation of RIPK3 and MLKL. Phosphorylated MLKL oligomers translocate to and perforate the plasma membrane, causing the extracellular release of danger-associated molecular patterns (DAMPs) including ATP. In a neighbouring cell (epithelial or immune cell), ATP activates purinergic receptor P2X7R to induce the assembly of NLRP3 inflammasome complex to facilitate the processing of pro-inflammatory cytokine IL-1β. ASC Apoptosis-associated speck-like protein containing a CARD, casp1 caspase-1, DAMPs Danger-associated molecular patterns, IL-1β Interleukin-1 beta, MLKL, mixed lineage kinase domain-like, p-MLKL Phosphorylated mixed lineage kinase-domain like, NLRP3 NLR family pyrin domain containing 3, P2X7R P2X purinoceptor subfamily 7, RIPK1 Receptor-interacting protein kinase 1, RIPK3 Receptor-interacting protein kinase 3, TNF-α Tumour necrosis factor-alpha, TNFR Tumour necrosis factor receptor. Created with Biorender.

The present study for the first time demonstrated concurrent activation of necroptosis and inflammasome in the small intestine following major surgery in the form of renal transplantation or renal ischaemia-reperfusion in rodents. Blocking necroptosis with the RIPK1 inhibitor Nec-1s or the MLKL inhibitor NSA effectively prevented inflammasome activation in the small intestinal cells. Taken together the current and our previously published work whereby traumatic surgery led to remote pulmonary and hepatic injury that was accompanied by increased TNF-α in the systemic circulation [15, 16, 28], the necroptosis-inflammasome axis may be therapeutically targeted to tackle postoperative multiorgan injury (Fig. 8).

Necroptosis has been implicated in the ischaemia-reperfusion injury of heart, brain and kidneys [10, 29–32], solid organ transplantation injury [15, 33, 34] and autoimmune diseases (e.g. arthritis [14], multiple sclerosis [35]). Necrostatin-1 (the predecessor RIPK1 inhibitor to Nec-1s) or genetic deletion of RIPK3 and/or MLKL were shown to be organ-protective and anti-inflammatory in these disease models, with the therapeutic benefits from MLKL depletion alone appeared to be less pronounced [36]. Gene knockout studies attributed necroptosis as a pro-inflammatory form of death. Animals with skin-specific or intestinal-specific knockout of caspase-8 or Fas-associated protein with death domain (FADD), i.e. strategies that impaired apoptosis, developed prominent tissue necroptosis and inflammation [12, 13, 37] and was reversed by RIPK3-KO. Nec-1 treatment [38] or RIPK3 deficiency [36] also protected against TNF-α induced systemic inflammatory response syndrome (SIRS).

Studies on macrophages concluded that necrosome directly activates the inflammasome complex to promote IL-1β release without inducing necroptosis or independent from MLKL. In an alternative model, it was suggested that phosphor-MLKL oligomers perforated plasma membrane to release intracellular contents as danger-associated molecular patterns (DAMPs) to target the DAMP receptors, subsequently activating the inflammasome complex [17, 18]. Herein we demonstrated that pMLKL-positive and NLRP3/caspase1-positive cells are in close proximity in the injured small intestines (Fig. 4), and that pMLKL and NLRP3 signals were either individually upregulated in neighbouring intestinal epithelial cells or co-localised within the same cell (Fig. 5). Moreover TLQ-induced extracellular ATP release was suppressed by MLKL inhibitor NSA (Fig. 6F). Our data suggests that postoperative small intestinal injury could encompass both paradigms of “necro-inflammation” and involves multiple cell types with heterogenous inflammasome activation pathways, potentially forming a positive amplification loop of injury and inflammation.

Intestinal dysfunction is a well-recognised severe complication in surgical and ICU patients and is associated with worse outcomes [4, 7, 39–42]. Clinical symptoms of intestinal injury/dysfunction manifest in a continuum of severity (from feeding intolerance to stress ulcer bleeding), and can be present in 20–60% of patients in critical care [43, 44]. Aside from its primary role in absorption, the intestines are also the largest immune organ within the body to accommodate ~40% of functional immune cells and represent a robust reservoir of pro-inflammatory cytokines (IL-1, IL-6, TNF-α). The “gut hypothesis” of multiorgan injury suggested the intestines may readily respond to perturbations of gut microbiota (commensal or pathogenic bacteria), ischaemia-reperfusion injury or direct surgical stress/trauma, and are capable of systemic propagation of local inflammation to affect distant organs [7, 40]. Our current finding on inflammasome activation in small intestines indicates the postoperative gut to be capable of initiating and amplifying inflammation. Moreover, our recent work demonstrated that surgery-induced gut barrier breakdown and gut microbiota dysbiosis predicted cognitive decline in patients with prodromal Alzheimer’s disease [42].

Different therapies have been trialled to preserve gut function in surgical and critically-ill patients [45, 46]. Our group and others have demonstrated the alpha2-adrenergic agonist dexmedetomidine to be highly cytoprotective, organ-protective and anti-inflammatory in ischemia-reperfusion injury and sepsis [47–50]. A small clinical study found that dexmedetomidine reduced intestinal injury marker diamine oxidase following liver resection [51]; future studies are necessary to ascertain whether dexmedetomidine improves postoperative intestinal function and reduces permeability with larger sample size, and to explore whether dexmedetomidine confers gut protection by inhibiting necroptosis and inflammasome activity as elucidated herein.

Our study is not without limitations. First, transgenic animals such as RIP knockout, MLKL knockout and NLRP3 knockout would be considered for further investigation as they are more specific than the inhibitors used in this study; still, NLRP3 detection in vivo and NLRP3 and p-MLKL determined in vitro pointed to the critical role of NLRP3 in regulated cell death exemplified by necroptosis. Second, we could not eliminate other detrimental effects associated with acute kidney or kidney graft injury on the gut, such as accumulation of toxic waste. Finally, the effects of allo-responses from kidney transplant on gut injuries remain unknow. However, these responses are normally initiated about a week after engraftment whilst our gut injury was determined after 24 h after surgery when allo-response has yet to occur. Despite these limitations, our work report herein encourages further studies in this area of research to enhance outcomes following major surgery in elderly and/or vulnerable patients.

## Conclusion

In summary, our study provides novel insight into necroptosis and inflammasome activation as the pathophysiological mechanism underlying small intestinal injury following surgery, whereby necroptosis may regulate inflammasome activity through ATP release in the intestinal epithelium (Fig. 8). Our study highlights the therapeutic potential from collective targeting of necroptosis and inflammasome in the management of MOD after surgery and in critical care setting.

## Materials & methods

### Animal surgery and treatment

In the present study, rat allograft kidney transplantation surgery and mouse renal ischaemia-reperfusion injury were used as two different models of laparotomic major surgery. Inbred rats and mice were purchased from Harlan, Bicester, UK and were kept in temperature- and humidity-controlled cages in a specific pathogen-free facility at Chelsea-Westminster Campus, Imperial College London. All animal procedures were carried out in accordance with the United Kingdom Animals (Scientific Procedures) Act of 1986.

Adult male rats aged 12 to 16 weeks and weighing 225–250 grs were used in the allograft kidney transplantation model, where kidney from Brown-Norway (BN, RT^1n^) rat was transplanted orthotopically into Lewis (LEW, RT1^1^) rat using conventional microvascular techniques under isoflurane anaesthesia (4% induction, 2% maintenance) as described before [15, 16, 28]. In the Brown-Norway donor rats, the left kidney, aorta and inferior vena cava were exposed to extract the graft to be flushed. Lewis recipient rats were randomly allocated to naïve control (no transplantation, *n* = 4), cold-ischaemia 0 h (CI0, *n* = 4) or cold-ischaemia 24 h (CI24, *n* = 4). The CI0 group was used to emulate live transplantation, whereby the recipient’s left kidney was removed and flushed, followed by anastomosis of the renal arteries, veins and ureters between the donor and the recipient, with total surgical ischaemia time limited to < 45 min. Recipient’s contralateral naïve kidney was removed immediately after surgery. Successful engraftment and perfusion are confirmed by an immediate colour change of the kidney graft and expansion of the renal arteries/veins. For the CI 24 h group that mimics delayed transplantation, donor’s left kidney was stored in 4 °C heparinised Soltran’s preservation solution for 24 h, prior to implantation into the recipient as described above. Additional CI24 groups were given a single dose of necrostatin-1s (1.65 mg/kg in 200 µL PBS, intraperitoneal, *n* = 4; Nec-1s, 17802, Cell Signaling Technology, MA, USA) or vehicle PBS (200 µL, intraperitoneal, *n* = 4) immediately after transplantation.

Adult male C57BL/6 mice weighing 20–25 grs were randomly divided into naïve control, sham surgery, renal ischaemia-reperfusion (I/R), or renal I/R + necrostatin-1s (*n* = 4–5 per group). I/R mice were anaesthetised by ketamine (100 mg/kg, i.p) and xylazine (10 mg/kg, i.p). After which, the left kidney pedicle was exposed and clamped for 30 min, followed by unclamping and closure of abdomen. The sham mice received incision of the abdomen only under anaesthesia.

In view of our previous studies on renal transplantation and remote organ injury [15, 16], a sample size of *n* = 4–5 was considered to be sufficient for the present study. For both renal transplantation and renal ischaemia/reperfusion procedures, randomisation was achieved using a computer-based random number generator. For each animal, two different investigators were involved, whereby the first investigator performed the surgical procedures +/− treatment based on the randomised numbers and was aware of the group allocations; the second investigator was responsible for harvesting samples for subsequent experiments and data analysis. As the NC animals did not receive laparotomic surgery, blinding was not feasible for the NC group.

All animals were given post-operative analgesics buprenorphine (0.1 mg/kg, subcutaneous) and carprofen (5 mg/kg, subcutaneous) daily for three days after the procedure. No animals died during the period of study.

### Cell culture

Human colonic epithelial cancer cells Caco-2 (European Collection of Authenticated Culture Collection) with small intestinal epithelium-like characteristics [52, 53] and human leukaemic U937 cells (ECACC) adopt the characteristics of mature macrophage [54] were used in the present study. Caco-2 was cultured in Dulbecco’s Modified Eagle Medium (DMEM) medium (10569010, Gibco, ThermoFisher Scientific, MA, USA) with non-essential amino acids (11140035, Gibco), 10% foetal calf serum (A3840401, Gibco) and 1 U/mL penicillin-streptomycin (10378016, Gibco) and were kept at 70–80% confluency. U937 were cultured separately in RPMI 1640 medium (61870036, Gibco) supplemented with 10% FCS and 1 U/mL antibiotics. All cultures were maintained in 5% CO_2_ balanced with air in an incubator at 37 °C.

### Cell treatment

Cells were treated with the following combination of compounds: recombinant human TNF-α (50 ng or 100 ng/mL; 210-TA-005, R&D, Abington, UK) and SMAC mimetic LCL-161 (100 nM; I1044, Cambridge, Cambridge Bioscience, UK), abbreviated as TL, T50 + L or T100 + L; TNF-α (50 ng or 100 ng/mL), LCL-161, and the pan-capsase inhibitor Q-VD-Oph (20 µM; 15260, Cayman Chemicals, MI, USA), abbreviated as TLQ, T50 + L + Q, or T100 + L + Q. Additional groups also received the RIPK1 inhibitor necrostatin-1s (Nec-1s, 20 µM; 17802, Cell Signaling Technology), abbreviated as TLQ+Nec-1s, T50 + L + Q+Nec-1s or T100 + L + Q+Nec-1s, or the MLKL-specific inhibitor necrosulfonamide (NSA, 2 µM; 5025/10, Tocris, Bristol, UK), abbreviated as TLQ + NSA, T50 + L + Q + NSA or T100 + L + Q + NSA. U937 cells were first differentiated with 100 ng/mL phorbol 12-myristate 13-acetate (PMA; P8139, Merck, Dorset, UK) for 24 h [55] prior to co-culturing with Caco-2 cells at a ratio of 1:4 (U937: Caco-2). For ATP release assay, all groups received ecto-ATPase inhibitor ARL67156 (200 μM, Tocris) to stabilise extracellular ATP. Cell were treated for 6 h, 18 h or 24 h prior to analysis, and the timepoints were chosen with reference to previous studies on necroptosis and/or inflammasome activities [14, 20, 26, 56].

### Immunofluorescent staining

On day 1 after surgery, rats and mice received ascending aortic perfusion with 4% paraformaldehyde (sc-281692, Santa Cruz). Small intestine samples were cryosectioned into 15 μm thick sections. Caco-2 cells were fixed with ice-cold 4% PFA for 10 min at 6 h or 24 h after treatments. Frozen sections and cells were blocked in 10% normal donkey serum in phosphate buffer saline with Triton-X (PBST) at room temperature for 1 h followed by overnight incubation at 4 °C in primary antibodies: rabbit monoclonal anti-P2X7 (1:500, ab259942, Abcam; Cambridge, UK), mouse monoclonal anti-NLRP3 (1:500, AG-20B-0014-C100, Adipogen; CA, US), rabbit polyclonal anti-caspase 1 (1:200, Ab1872, Abcam), mouse monoclonal anti-RIP1 (1:250, sc-133102; Santa Cruz), mouse monoclonal anti-RIP3 (1:250, sc-374639, Santa Cruz), and rabbit monoclonal anti-mouse phosphorylated MLKL (1:500, 37333, Cell Signalling Technology). On day 2, tissue and cell samples were incubated in secondary antibodies for 1 h at room temperature: donkey anti-rabbit IgG AlexaFluor488 (1:500, A-21206, ThermoFisher; Waltham, MA, US), donkey anti-rabbit IgG AlexaFluor568 (1:500, A10042, ThermoFisher), donkey anti-mouse IgG AlexaFluor488 (1:500, A32766, ThermoFisher), and donkey anti-mouse IgG AlexaFluor568 (1:500, A10037, ThermoFisher). Tissues or cells were counterstained with DAPI-mounting medium (H-1200, Vector Laboratories; Burlingame, CA, US) and viewed under Zeiss AxioObserver 7 microscope. Representative images were captured and fluorescence intensities were semi-quantified using ImageJ (National Institutes of Health, Bethesda, MD). Fluorescence intensity from different groups were expressed as relative changes to the naïve controls.

### Haematoxylin and eosin staining

On day 4, PFA-fixed small intestine specimens were dehydrated in ethanol and xylene before paraffin embedment, and were cut into 5 um thick sections for haematoxylin and eosin staining. Slides were viewed under Olympus BX4 microscope and representative images for each group were taken.

### Western blot

Tissue and cell samples were homogenised with cell lysis buffer (Cell Signaling Technology) supplemented with protease inhibitor (11873580001, Roche, Switzerland) and phosphatase inhibitor (4906845001, Roche). Protein contents were quantified using Bio-rad Protein Assay (Bio-rad, Hertfordshire, UK) and were denatured at 95 °C before gel electrophoresis. Protein bands were transferred onto nitrocellulose membrane using iBlot system (Invitrogen) following manufacturer’s instruction. The membrane was blocked in 5% non-fat milk followed by overnight incubation in primary antibodies: rabbit anti-P2X7 (1:1000, ab259942, Abcam), mouse anti-NLRP3 (1:1000, AG-20B-0014-C100, Adipogen), rabbit anti-ASC (1:500, Santa Cruz), rabbit anti-caspase1 (1:500, Ab1872, Abcam), mouse anti-RIP1 (1:500, sc-133102, Santa Cruz), rabbit anti-mouse phosphorylated MLKL (1:1000, 37333, Cell Signaling Technology), mouse anti-interleukin 1β (1:1000, 12242, Cell Signaling Technology), rabbit anti-cleaved interleukin 1β (1:1000, 83186, Cell Signaling Technology), and mouse anti-GAPDH (1:20,000, MAB374, Merck Millipore). On day 2, the membrane was incubated in donkey anti-rabbit HRP-conjugated IgG (1:3000; 7074, Cell Signaling Technology) or donkey anti-mouse HRP-conjugated IgG (1:3000; 7076, Cell Signaling Technology) for 1 h at room temperature. The blots were developed with ECL system (sc-2048; Santa Cruz) and visualised with GeneSnap (Syngene, Cambridge, UK). Densitometry analyses were performed with ImageJ, and protein band intensity was standardised to GAPDH and expressed as relative changes to naïve control.

### Cell counting Kit-8 assay

Caco2 cells were seeded into 96-well plates and cultured until confluent ( ~ 1 x 10^4^ cells/well). At 6, 18 or 24 h after treatments (*n* = 5–7/group), 10 μL of CCK-8 assay solution (96992, Merck) was added into each well to react for 2 h in dark. Cell viability was quantified by measuring the absorbance at a wavelength of 450 nm using microplate reader (ELx800 Bio-Tek; Santa Clara, CA, US). Cell viability was expressed as the percentage of NC.

### Propidium Iodide/Hoechst 33342 staining

Caco-2 cells were stained with propidium iodide (5 µg/mL; P3566, Invitrogen) and Hoechst 33342 (5 µg/mL; H3570, Invitrogen) dyes for 5 min in dark at 37 °C, whereby PI only stains the nuclei of dead cells and Hoechst 33342 stains nuclei of all cells, before viewing under Olympus BX4 microscope.

### ATP release assay

Caco-2 cells (*n* = 8) were seeded into 24-well corning plates and exposed to treatments for 6 h. 100 μL of medium from each well was collected and extracellular ATP release was quantified with ATPLite Luciferase ATP Detection Assay System following manufacturer’s instructions (6016943, Perkin Elmer, Waltham, MA, USA) and with a luminescence plate reader (FLx800, Bio-Tek).

### Statistical analysis

Immunofluorescent intensity, western blot and ATP release assay were analysed with Kruskal-Wallis test and corrected by Dunn’s post-hoc test, as these datasets did not pass the Kolmogorov-Smirnov normality test or the n number was too small to be tested for normal distribution. Data were expressed as mean ± standard deviation plus dot plot. CCK-8 assay was analysed by one-way ANOVA and corrected by Tukey’s post-hoc test as the data passed normality test. Data were expressed as mean ± standard deviation (SD) plus dot plot. A two-tailed *P*-value less than < 0.05 was considered to be of a statistical significance. All statistically analyses were done with GraphPad Prism version 5 (La Jolla, CA, USA).

### Supplementary information

Supplementary material

## Data Availability

All data generated or analysed during this study are included in this published article and its supplementary information files.
