# Supplementary material for: Regulated cell death and inflammasome activation in gut injury following traumatic surgery in vitro and in vivo: implication for postoperative death due to multiorgan dysfunction
Source: Cell Death Discov. 2023 Nov 7;9:409. doi: 10.1038/s41420-023-01647-z (PMC10630406; doi:10.1038/s41420-023-01647-z)

Original WB

Supplementary File 1

Figure 1D

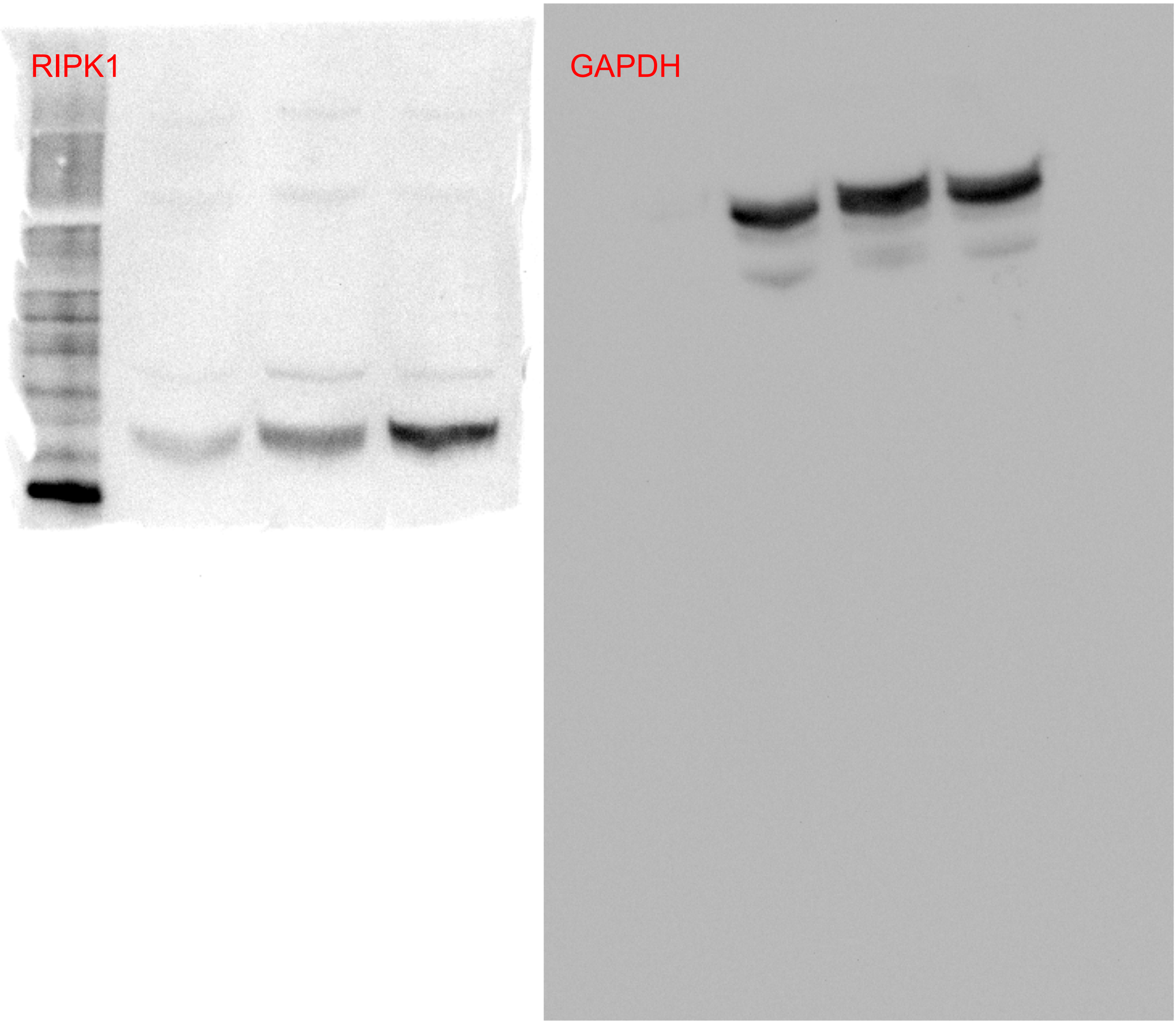

Supplementary File 2

Figure 2E

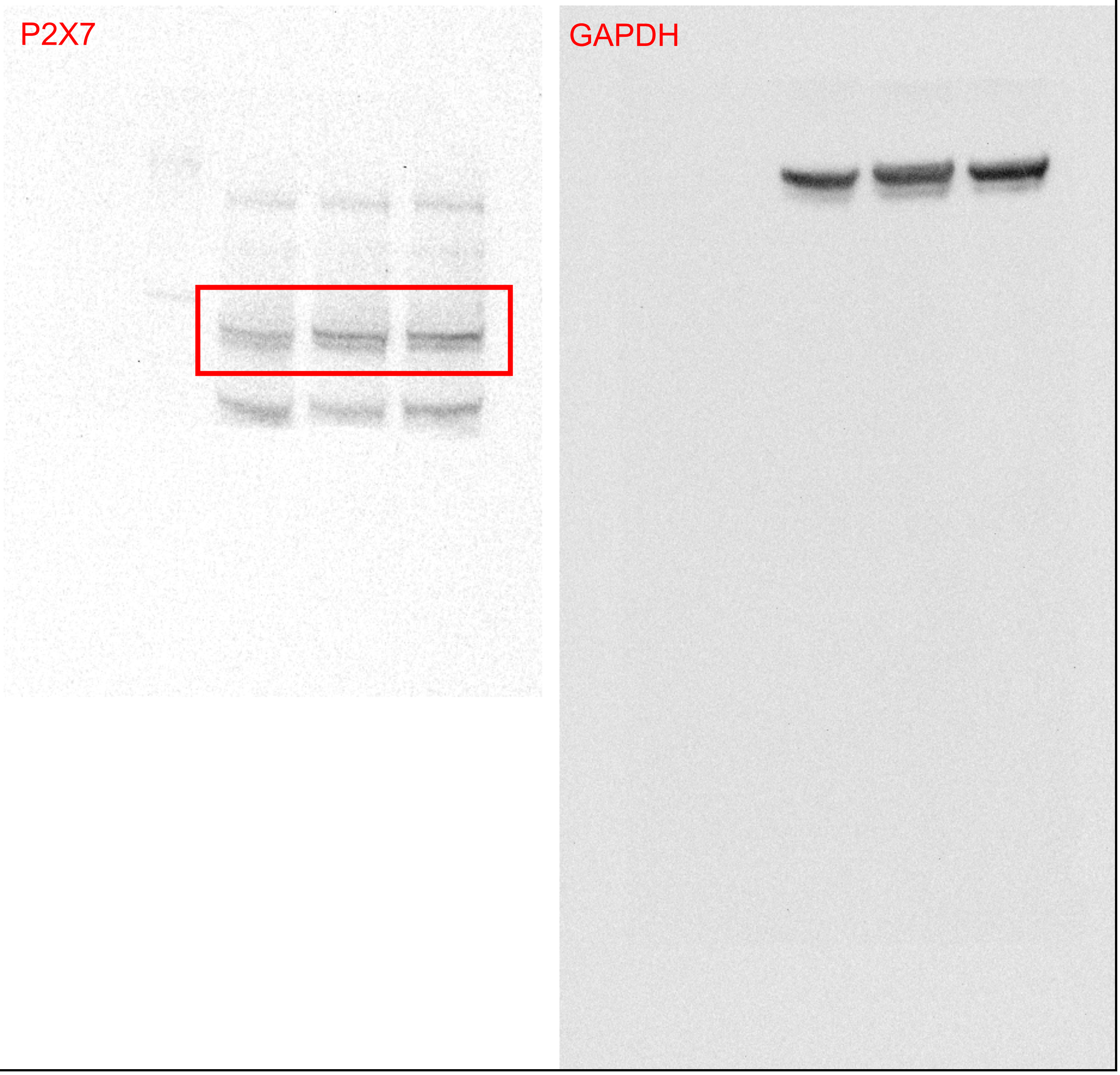

Supplementary File 3

Figure 2F

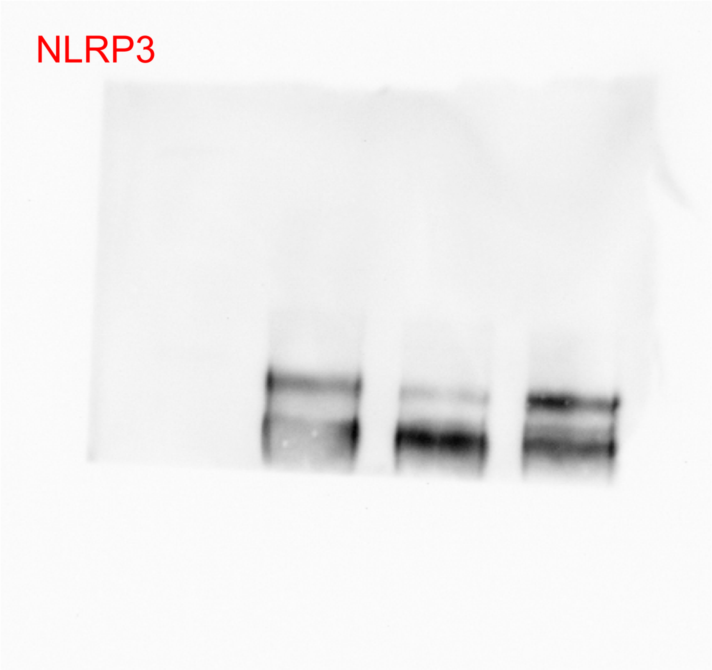

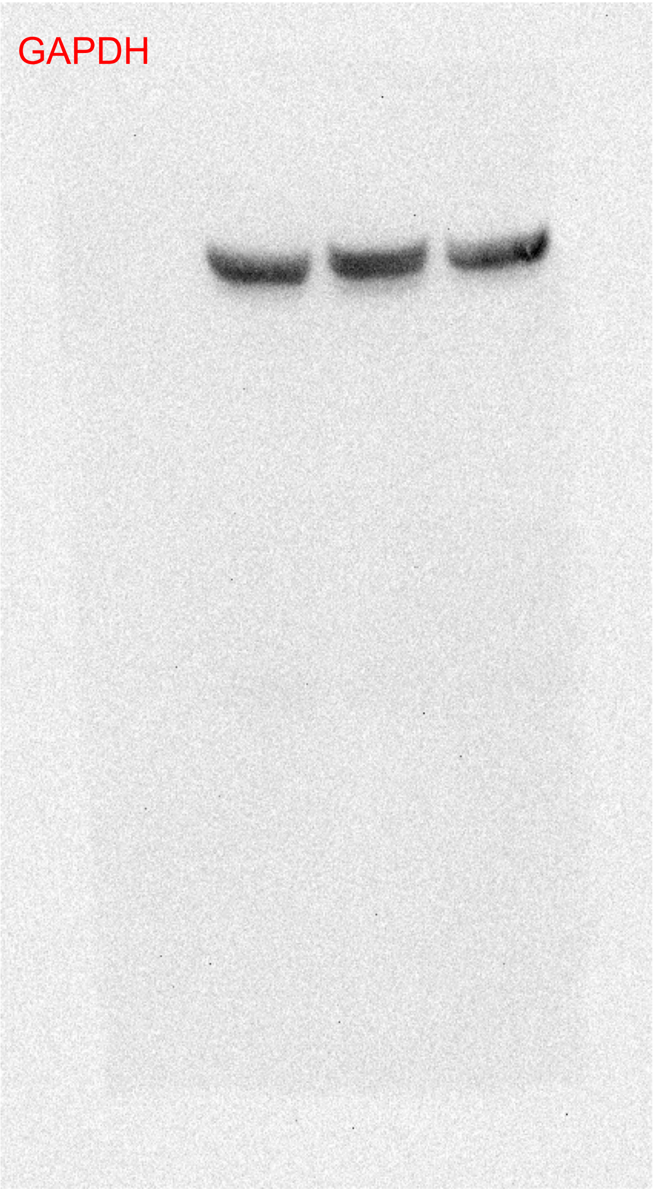

Supplementary File 4

Figure 2F

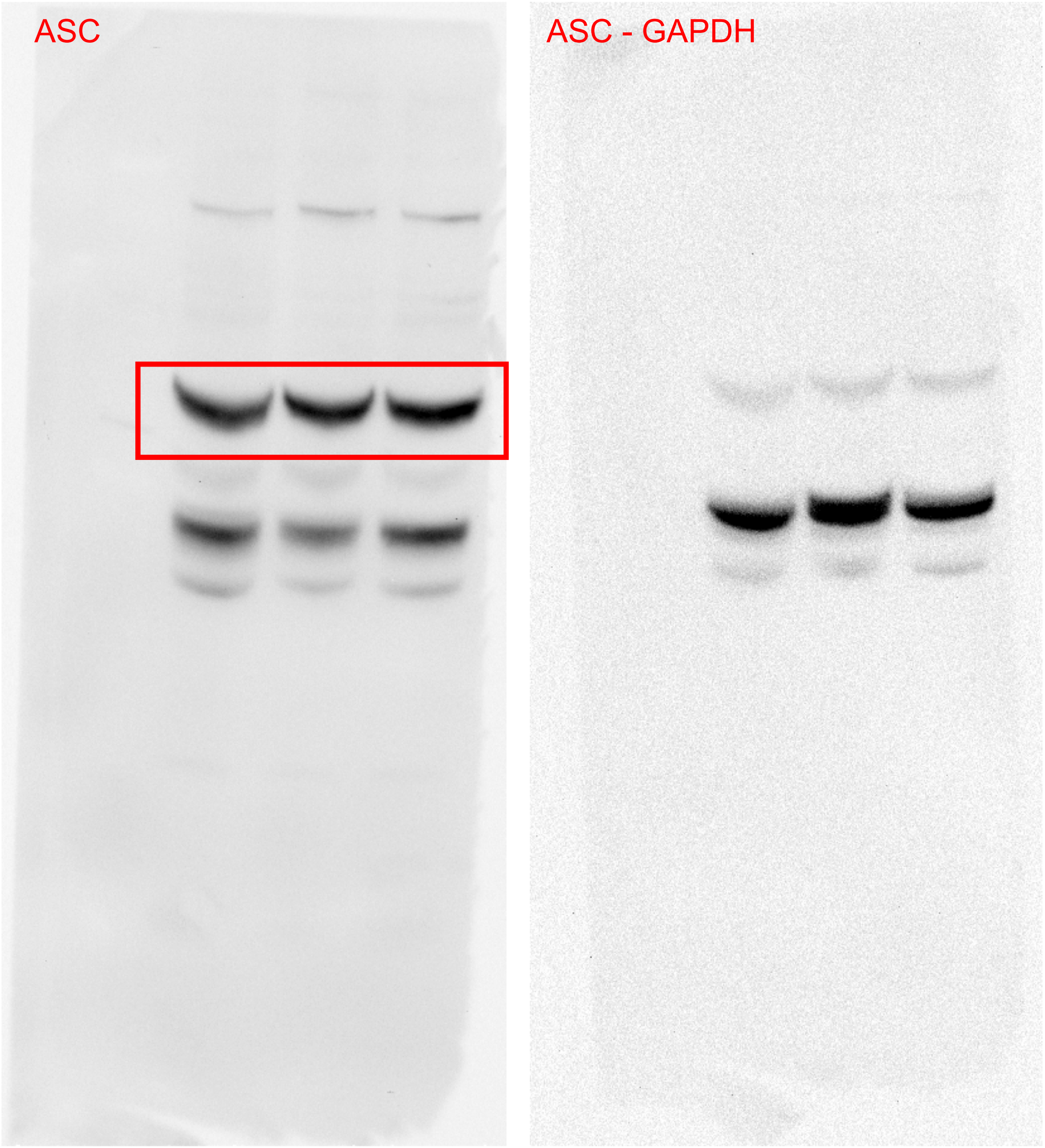

Supplementary File 5

Figure 2G

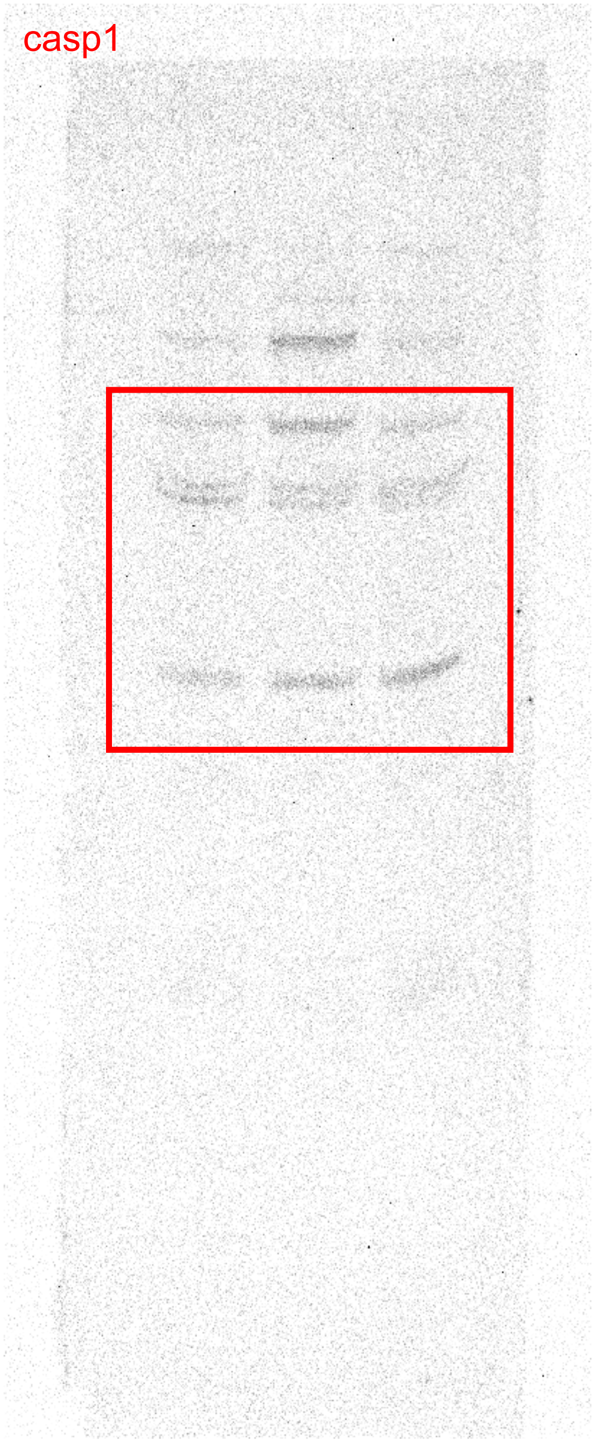

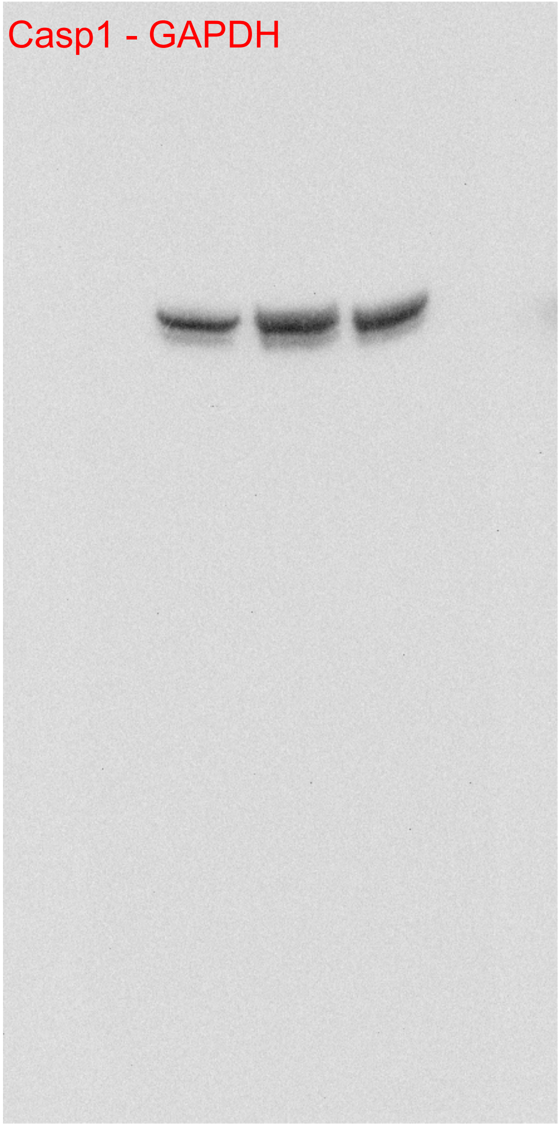

Supplementary File 6

Figure 3D

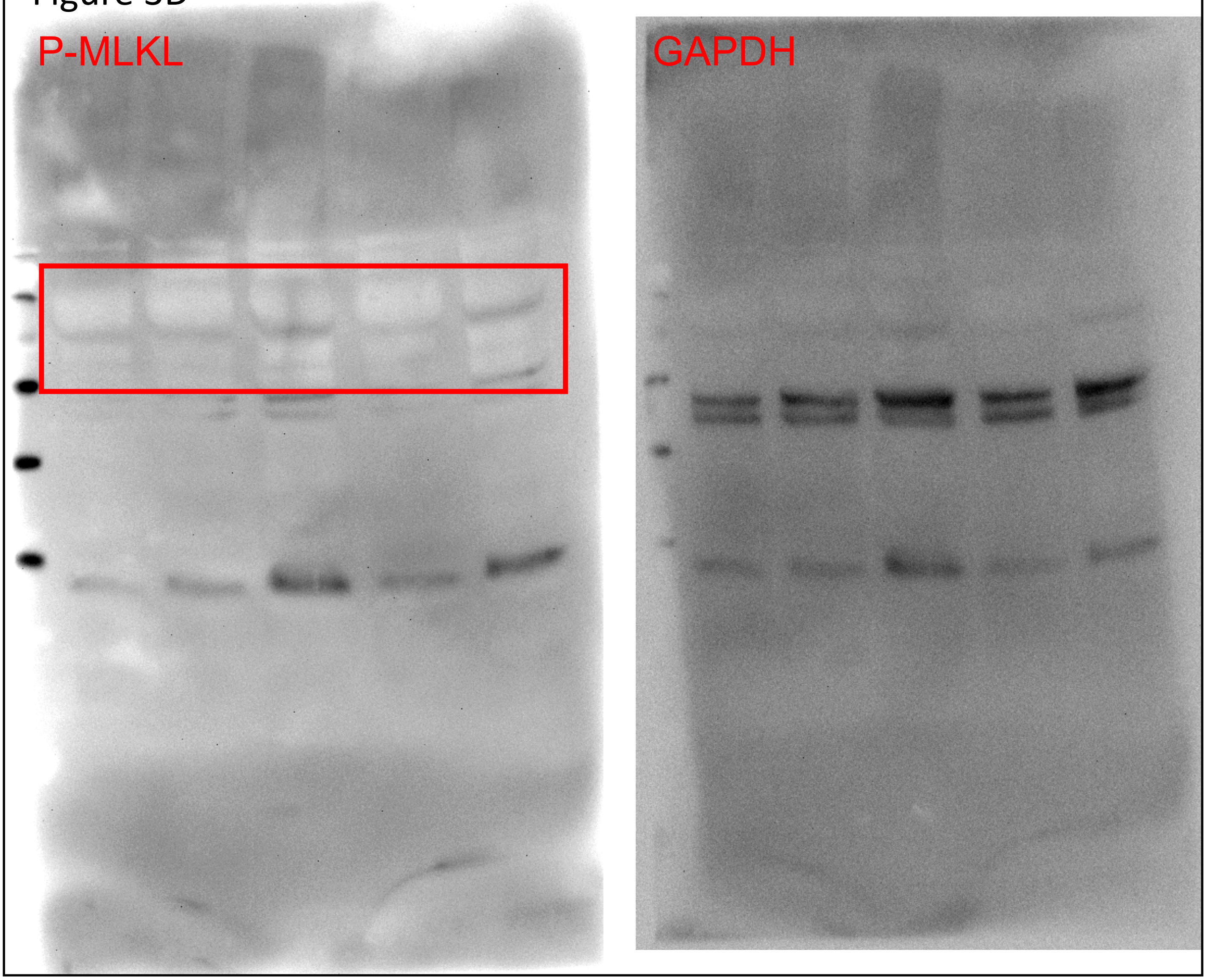

Supplementary File 7

Figure 3F

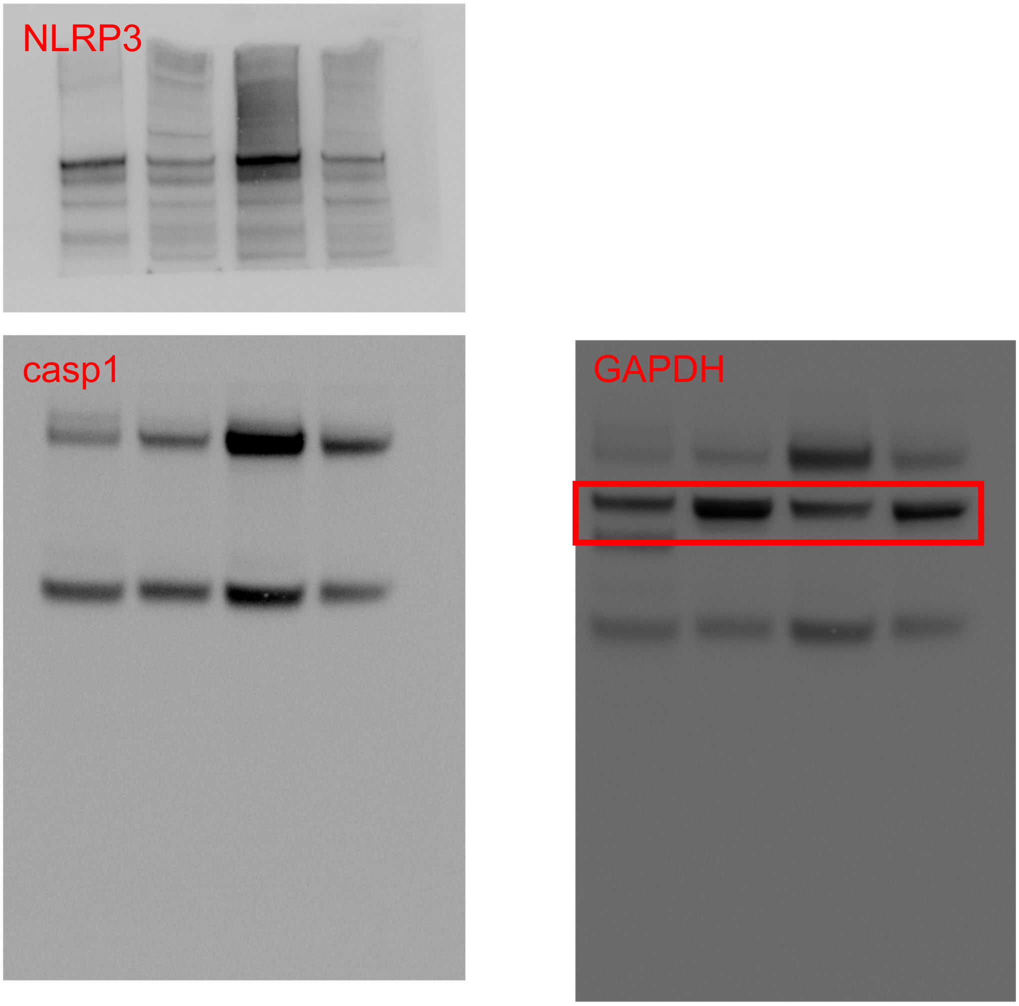

Supplementary File 8

Figure 7A

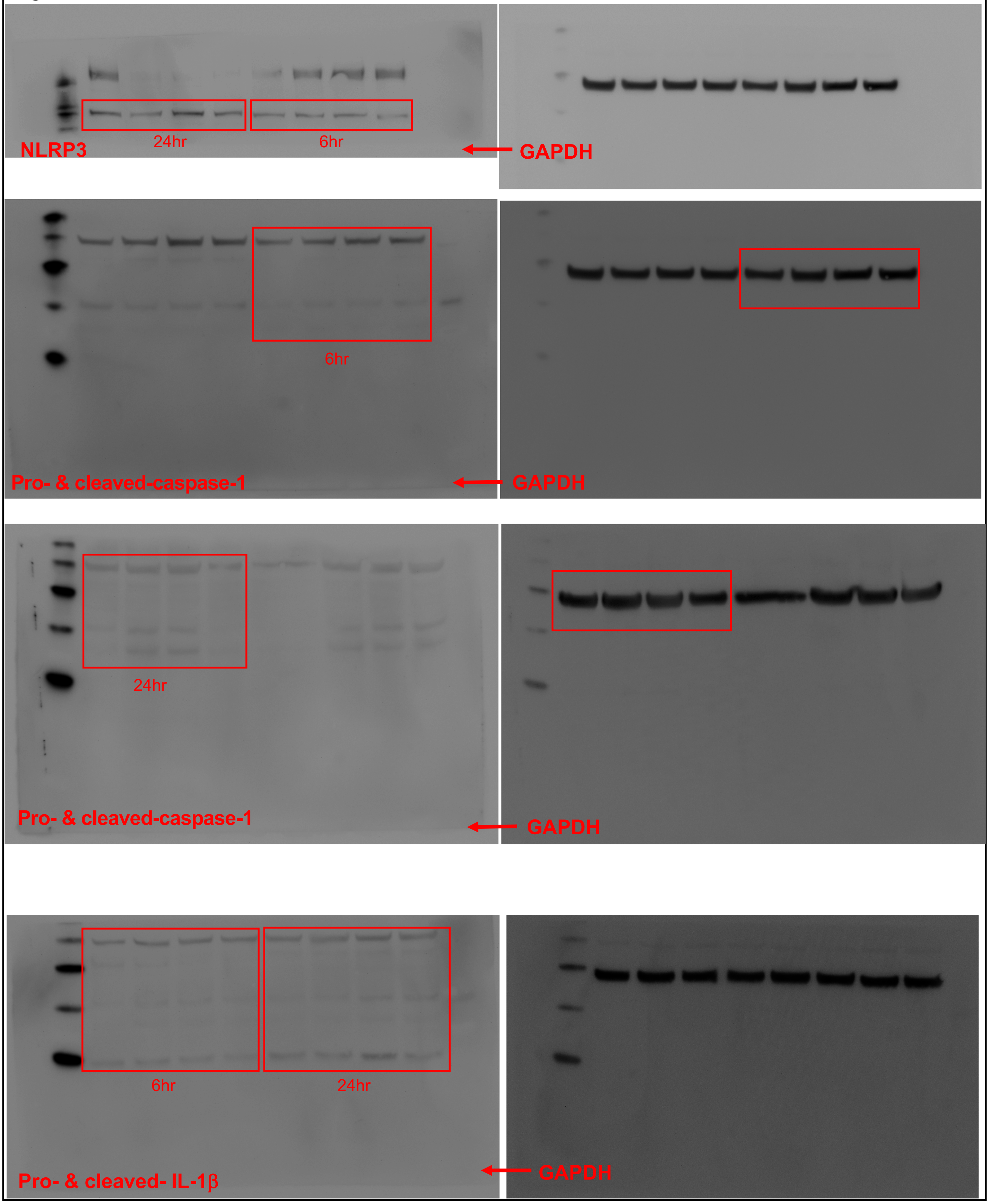

Supplementary File 9

Figure 7B

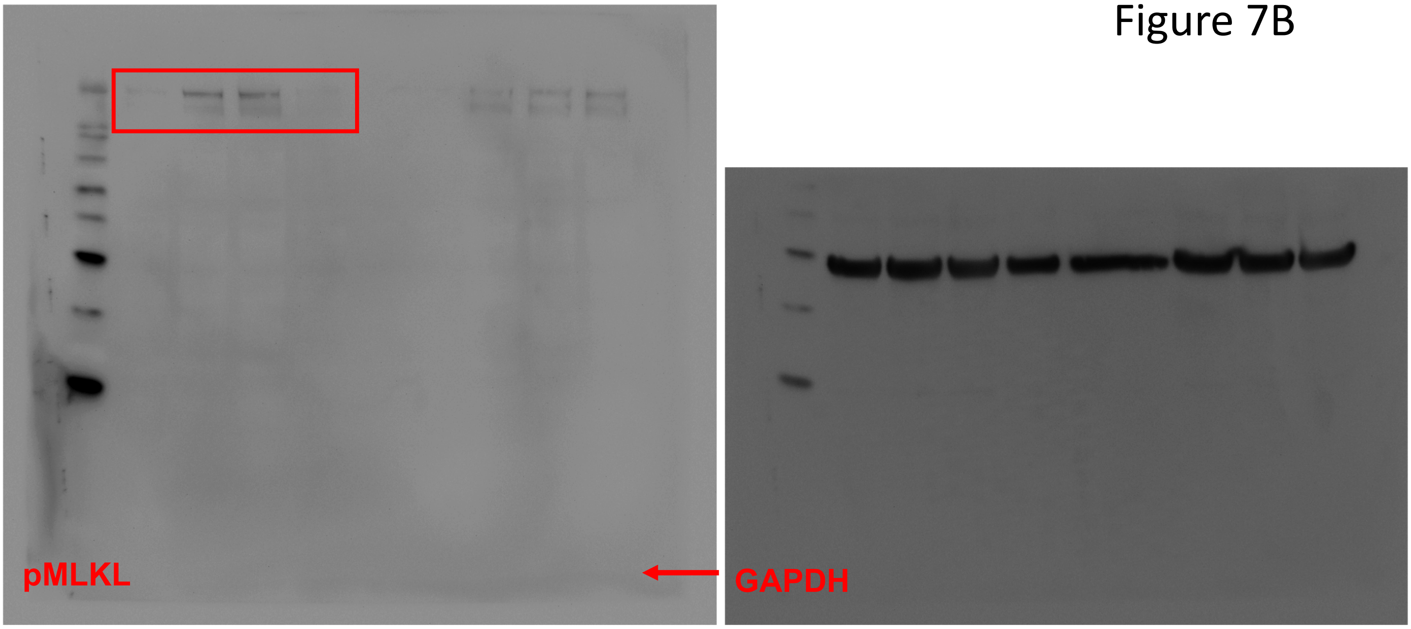

*Same membrane as caspase-1 from Fig.7A

Supplementary File 10

Figure 7E

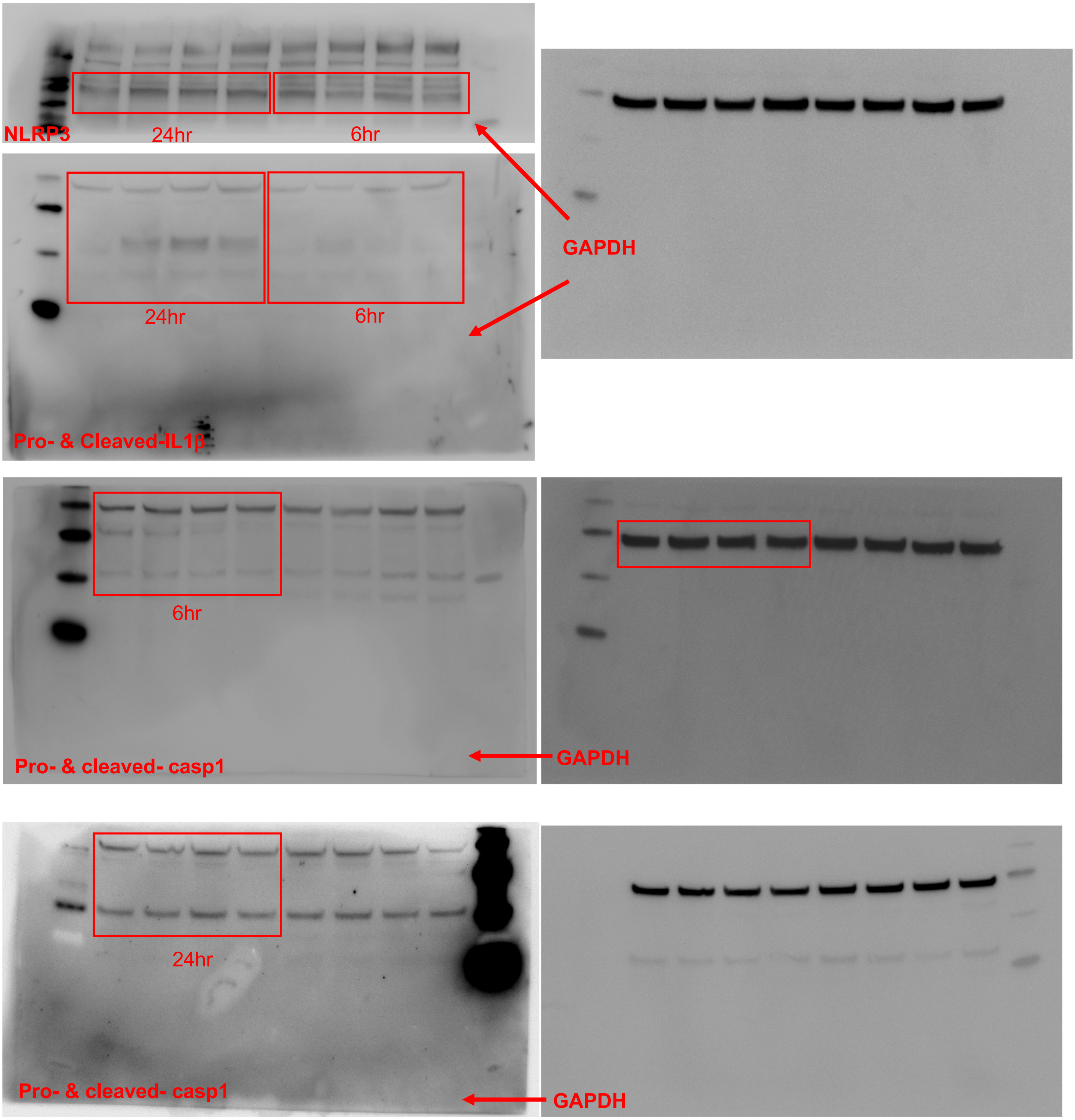

Supplement: Supplementary file 1 — Supplementary material [file 41420_2023_1647_MOESM1_ESM.pdf]
